# Changes in blood pressure following the relocation of individuals to well-insulated and well-ventilated apartments building

**DOI:** 10.1038/s41440-026-02673-x

**Published:** 2026-05-13

**Authors:** Hironori Nakagami, Ryoichi Ohara, Atsushi Iwamae

**Affiliations:** 1 Department of Health Development and Medicine, Graduate School of Medicine, The University of Osaka, Suita, Osaka Japan; 2 Department of Geriatric and General Medicine, Graduate School of Medicine, The University of Osaka, Suita, Osaka Japan; 3 Department of Architecture, Kindai University Faculty of Architecture, Higashi Osaka, Osaka Japan

**Keywords:** Morning hypertension, Indoor environment, Apartment, Digital hypertension, Net zero energy house

## Abstract

To explore the effect of the indoor environment on blood pressure (BP) at home, individuals moving into newly constructed, well-insulated, and well-ventilated apartment buildings were targeted in this study. The BP of the participants was measured in February for two consecutive years before and after the participants moved. The analysis included 179 and 178 individuals with morning and evening BP measurements, respectively. No overall change in BP was observed before and after the participants moved. In the subgroup analysis, in the antihypertensive treatment group, the systolic and diastolic morning BP decreased by ~7 and 5 mmHg, respectively. In the hypertension and elderly participant groups, the systolic and diastolic BP decreased by ~4–6 mmHg and 2–4 mmHg, respectively. This reduction in BP correlated with the subjective temperature in the bedroom. These results demonstrate the impact of the indoor environment on BP control in apartments.

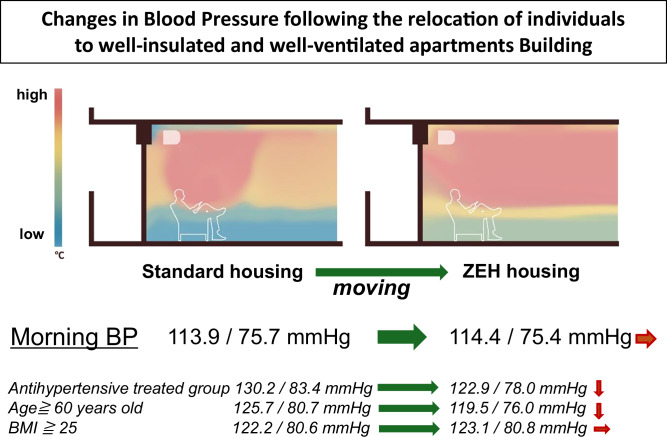

## Introduction

An increase in blood pressure (BP) in response to cold leads to increased mortality from cardiovascular disease in the winter [1–3]. Recently, Net Zero Energy House (ZEH) was proposed to refer to a residence that aims to achieve a net zero balance in annual primary energy consumption. This is accomplished by significantly improving the thermal insulation performance of the building envelope and introducing high-efficiency equipment systems to maintain quality of the indoor environment. However, there are few recent reports on the relationship between the indoor environment and BP. The focus of this study was individuals moving into newly constructed, well-insulated, and well-ventilated apartment buildings. Home BP measurements were taken in February prior to relocation and one year later in February after moving. Changes attributable to the residential environment were evaluated on the basis of these BP measurements.

Point of view

- Clinical relevance
  After the relocation to well-insulated and well-ventilated apartments building, the systolic and diastolic BP significantly decreased in subgroup analysis with high BP.
- Future direction
  Improving living conditions was associated with a reduction in BP during the winter season, which supports the impact of the indoor environment on BP control.
- Consideration for the Asian population
  Climate change is accelerating in Asia as well, and improving the indoor environment is increasingly important for BP management.

## Methods

### Study design

This was a prospective observational study, and the study protocol was approved by the IRB of Kinki University (No. 2023-4). Participants were selected from prospective residents of apartments developed by DAIKYO or ANABUKI CONSTRUCTION with written informed consent.

### Measurement of vital data and collection of survey questionnaires

Home BP was measured with an upper arm blood pressure monitor (HEM-7126, OMRON). The measurement period was 3 weeks in February for two consecutive years, and measurements were taken twice each after the participants woke up and before the participants went to bed. The questionnaire surveys on the perceived room temperature on a three-point scale: 1 = cold, 2 = slightly cold, 3 = not cold were conducted before and after the participants moved.

### Study endpoint

The average and standard deviation (SD) values of home systolic BP (SBP) and diastolic BP (DBP) were calculated and analyzed before and after relocation. Furthermore, we conducted subgroup analyses by sex, hypertension status, antihypertensive treatment status, age, and obesity status. As a second endpoint, we examined the association between subjective changes in bedroom temperature and BP.

### Statistical analysis

A paired *t*-test was performed to determine significant differences between groups (Microsoft Excel 365).

## Results and conclusion

**Fig. 1:**
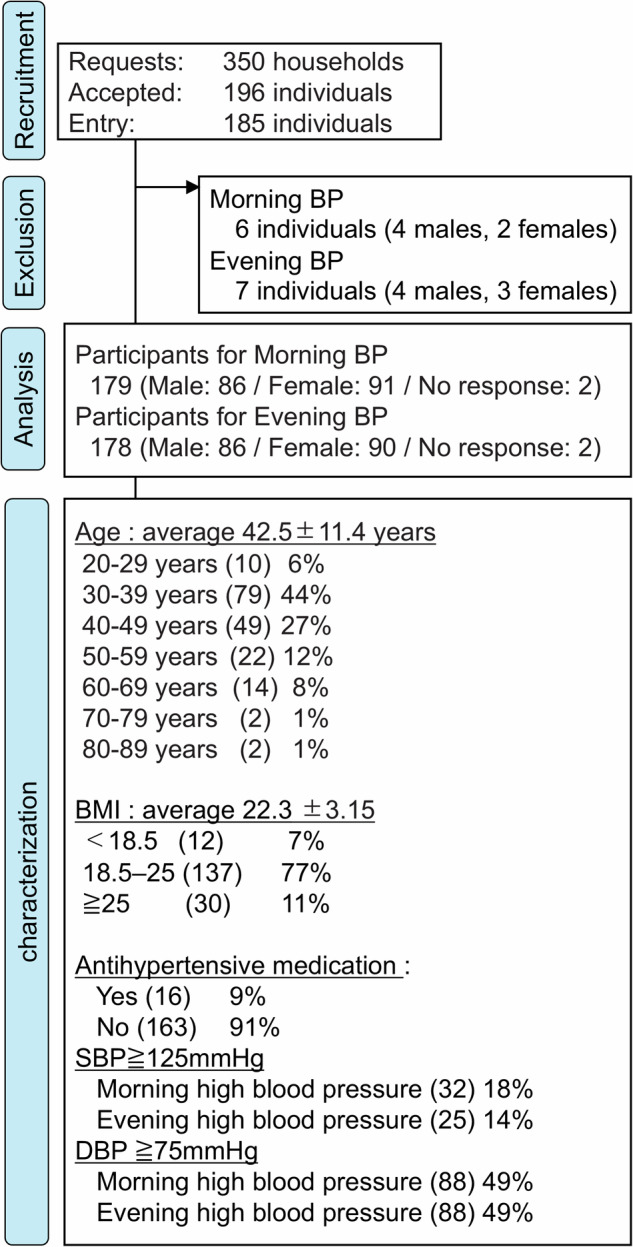
Study design. Among the 350 households, 196 individuals were accepted, and 185 individuals were enrolled. We analyzed the morning and evening BP of 179 and 178 participants, respectively. The average age of the participants was 42.5 years, and the average BMI was 22.3. The antihypertensive medication group accounted for 9% of the participants. The morning and evening systolic hypertension groups accounted for 18 and 14% of the participants, respectively, and the morning and evening systolic hypertension groups accounted for 49% of the participants, respectively. BP blood pressure, BMI body mass index, SBP systolic blood pressure, DBP diastolic blood pressure

In this study, 185 individuals were included in the analysis (Fig. 1). We analyzed the morning and evening BP of 179 and 178 participants, respectively. The average age of the participants was 42.5 years, with aged 60 years or older accounting for only 10% of the study population. Obese patients with a BMI of 25 or higher accounted for 11% of the participants, and the antihypertensive treatment group, in which the participants were prescribed blood pressure medication, constituted only 9%. Participants with high blood pressure with a SBP ≧125 mmHg or a DBP ≧75 mmHg accounted for 18 and 49% in the morning measurement group and 14 and 49% in the evening measurement group.

As shown in Table 1, no overall changes in morning SBP or DBP were observed, although men tended to have slightly higher blood pressure than women. Therefore, a subgroup analysis was performed to identify the population with high BP. Compared with the overall population, in the antihypertensive treatment group taking medication, the average BP was ~16 mmHg higher for SBP and ~8 mmHg higher for DBP. In this group, the SBP or DBP significantly decreased by ~7 or 5 mmHg, respectively, after the participants moved. In the group not taking antihypertensive medication, blood pressure had risen by ~1 mmHg, but this change remained within the normal range. Compared with the overall population, in the hypertension group and the elderly group (participants aged 60 or 65 years and older), the SBP or DBP was ~11–22 mmHg or 5–16 mmHg higher, respectively. The average BP significantly decreased after the participants relocated, with the SBP or DBP decreasing by ~4–6 mmHg or 2–4 mmHg, respectively. Many of these differences were statistically significant. In the group with normal blood pressure and the younger group (participants aged less than 60 or 65 years), blood pressure had risen by ~1 mmHg, but this change remained within the normal range. Interestingly, compared with the overall population, obese individuals with a BMI of 25 or higher had higher blood pressure. However, little difference in blood pressure was observed before and after relocation in this group. Similar results were observed for evening BP (Supplementary Table 1). The participants who took antihypertensive medication showed a marked change in blood pressure before and after the participants moved (~7 mmHg for SBP and 5 mmHg for DBP). Furthermore, BP changes before and after moving were compared with subjective temperature changes in the bedroom (Supplementary Fig. 1). In the overall analysis, the subjective room temperature tended to mildly increase from 1.94 to 2.22. However, in the antihypertensive treatment group, the subjective room temperature increased significantly from 1.63 to 2.63. In contrast, in the group with a BMI of 25 or higher, the change in the subjective room temperature was mild, from 1.85 to 2.14.

**Table 1:** Summary of morning BP before and after moving

Morning SBP				
Variables	*N*	Before	After	*P* value
Total	179	113.9 ± 13.6	114.4 ± 13.3	0.403
Male	86	121.0 ± 13.0	120.6 ± 13.4	0.675
Female	91	107.3 ± 13.1	108.7 ± 10.4	0.083
Antihypertensive medication	16	130.3 ± 13.0	122.9 ± 11.9	0.018*
No antihypertensive medication	163	112.3 ± 12.6	113.6 ± 13.2	0.026*
SBP ≧ 125 mmHg	32	135.9 ± 9.9	131.3 ± 14.4	0.020*
SBP < 125 mmHg	147	109.1 ± 8.7	110.7 ± 9.8	0.005*
Age ≧ 65 years old	11	128.9 ± 12.2	123.0 ± 13.6	0.137
Age < 65 years old	168	112.9 ± 13.1	113.8 ± 13.1	0.116
Age ≧ 60 years old	18	125.7 ± 17.4	119.5 ± 14.7	0.032*
Age < 60 years old	161	112.6 ± 12.5	113.8 ± 13.1	0.030*
BMI ≧ 25	30	122.2 ± 11.4	123.1 ± 12.4	0.484
BMI < 25	149	112.2 ± 11.4	112.7 ± 12.4	0.533
Morning DBP				
Total	179	75.7 ± 9.8	75.4 ± 10.0	0.532
Male	86	80.4 ± 9.9	79.5 ± 10.5	0.249
Female	91	71.3 ± 7.5	71.6 ± 7.7	0.565
Antihypertensive medication	16	83.4 ± 7.5	78.0 ± 6.1	0.003*
No antihypertensive medication	163	74.9 ± 9.7	75.2 ± 10.2	0.614
DBP ≧ 75 mmHg	88	83.2 ± 7.6	81.5 ± 9.4	0.011*
DBP < 75 mmHg	91	68.4 ± 5.0	69.6 ± 6.9	0.028*
Age ≧ 65 years old	11	82.6 ± 5.3	77.9 ± 8.3	0.024*
Age < 65 years old	168	75.2 ± 9.9	75.3 ± 10.1	0.976
Age ≧ 60 years old	18	80.7 ± 10.3	76.0 ± 9.0	0.005*
Age < 60 years old	161	75.1 ± 9.6	75.4 ± 10.1	0.624
BMI≧25	30	80.5 ± 9.6	80.8 ± 11.1	0.759
BMI < 25	149	74.7 ± 9.6	74.3 ± 9.4	0.447

The limitations of this study include the following. Since the majority of the cohort consisted of young individuals with normal BP, the subgroup analysis of the antihypertensive treatment group involved a small number of participants, making it difficult to perform an adequate statistical analysis. Information regarding antihypertensive medications is important, and we collected data on the number of types of medications each patient was taking. Twelve patients showed no change in the number of medications before and after moving, three patients were taking one fewer type, and information was unavailable for one patient. Therefore, it appears there was no significant impact on lowering blood pressure.

The Japanese Guidelines for the Treatment of Hypertension state that lifestyle guidance has been shown to lower BP includes reducing salt intake, taking potassium, maintaining a healthy body weight, exercising, and drinking less alcohol [4]. A meta-analysis revealed that reducing sodium intake was associated with a 2.64 mmHg reduction in SBP in office environments [5]; furthermore, increasing potassium intake was associated with 4.25 and 2.53 mmHg reductions in SBP and DBP, respectively [6]. Weight loss was associated with an ~1.1 mmHg reduction in SBP per kg of body weight [7], and a 2.27 reduction in BMI was associated with 5.79 and 3.36 mmHg decreases in SBP and DBP, respectively [8]. Furthermore, exercise, especially aerobic endurance exercise, was associated with an average reduction of 4.25 and 2.53 mmHg in SBP and DBP, respectively [9]. In this study, the SBP decreased by ~7 mmHg and the DBP decreased by ~5 mmHg after moving in the antihypertensive treatment group. While direct comparisons are not possible, the results suggest that improving the living environment is associated with a similar reduction in BP as lifestyle modifications. In the literature concerning the association between indoor temperature and BP, Saeki et al. reported that the daytime SBP increased by 0.44 mm Hg per 1 °C decrease in room temperature [10]. Umishio et al. reported that morning SBP was correlated with changes in the indoor temperature (8.2 mm Hg increase/10 °C decrease) [11]. Interestingly, obese individuals with a BMI of 25 or higher showed little change in blood pressure before and after moving, despite having higher average blood pressures. This result suggests that obese individuals may be more tolerant of temperature changes. A previous report revealed that the association between seasonal differences and BP was stronger in older people and among individuals with lower BMIs [12].

Climate change is accelerating in Asia as well. The results of this study demonstrated that improving living conditions was associated with a reduction in BP at home during the winter season, which supports the impact of the indoor environment on BP control.

## Supplementary information

Supplementary Table 1

Supplementary Figure 1
