# Supplementary material for: Changes in blood pressure following the relocation of individuals to well-insulated and well-ventilated apartments building
Source: Hypertens Res. 2026 May 13;49(7):2123–7. doi: 10.1038/s41440-026-02673-x (PMC13333491; doi:10.1038/s41440-026-02673-x)
Supplement: Supplementary file 1 — Supplementary Table 1 [file 41440_2026_2673_MOESM1_ESM.docx]

Supplement Table 1 Summary of evening BP before and after moving

Evening SBP

Variables N Before After P value

Total 178 112.1±12.9 112.9±12.3 0.198

Male 86 118.2±12.0 118.6±11.7 0.711

Female 90 106.2±10.8 107.5±10.2 0.086

Antihypertensive medication 16 126.7±13.8 119.4±9.8 0.017*

No antihypertensive medication 162 110.6±11.8 112.2±12.3 0.009*

SBP≧125mmHg 25 134.5±8.5 129.5±11.5 0.025*

SBP<125mmHg 153 108.4±9.2 110.2±10.1 0.005*

Age≧65 years old 11 124.7±12.6 118.0±8.8 0.086

Age<65 years old 167 111.2±12.5 112.5±12.4 0.036*

Age≧60 years old 18 120.6±18.0 114.5±11.9 0.026*

Age<60 years old 160 111.1±11.8 112.7±12.3 0.010*

BMI≧25 30 120.8±11.2 121.4±11.0 0.687

BMI<25 148 110.3±12.5 111.1±11.8 0.222

Evening DBP

Variables N Before After P value

Total 178 72.9±8.8 73.3±9.1 0.468

Male 86 76.6±8.8 76.9±9.3 0.634

Female 90 69.6±7.5 69.9±7.5 0.510

Antihypertensive medication 16 79.2±8.7 74.3±6.7 0.007*

No antihypertensive medication 162 72.3±8.6 73.1±9.3 0.060

DBP≧75mmHg 88 79.2±7.4 78.5±8.4 0.307

DBP<75mmHg 90 66.8±4.9 68.1±6.4 0.013*

Age≧65 years old 11 79.1±7.7 73.7±6.6 0.006*

Age<65 years old 167 72.5±8.8 73.2±9.2 0.118

Age≧60 years old 18 76.5±10.7 71.8±7.0 0.005*

Age<60 years old 160 72.5±8.5 73.4±9.3 0.047*

BMI≧25 30 77.5±9.2 78.2±10.2 0.356

BMI<25 148 72.0±8.5 72.2±8.5 0.662
