# Supplementary figures and images for: Changes in blood pressure following the relocation of individuals to well-insulated and well-ventilated apartments building
Source: Hypertens Res. 2026 May 13;49(7):2123–7. doi: 10.1038/s41440-026-02673-x (PMC13333491; doi:10.1038/s41440-026-02673-x)

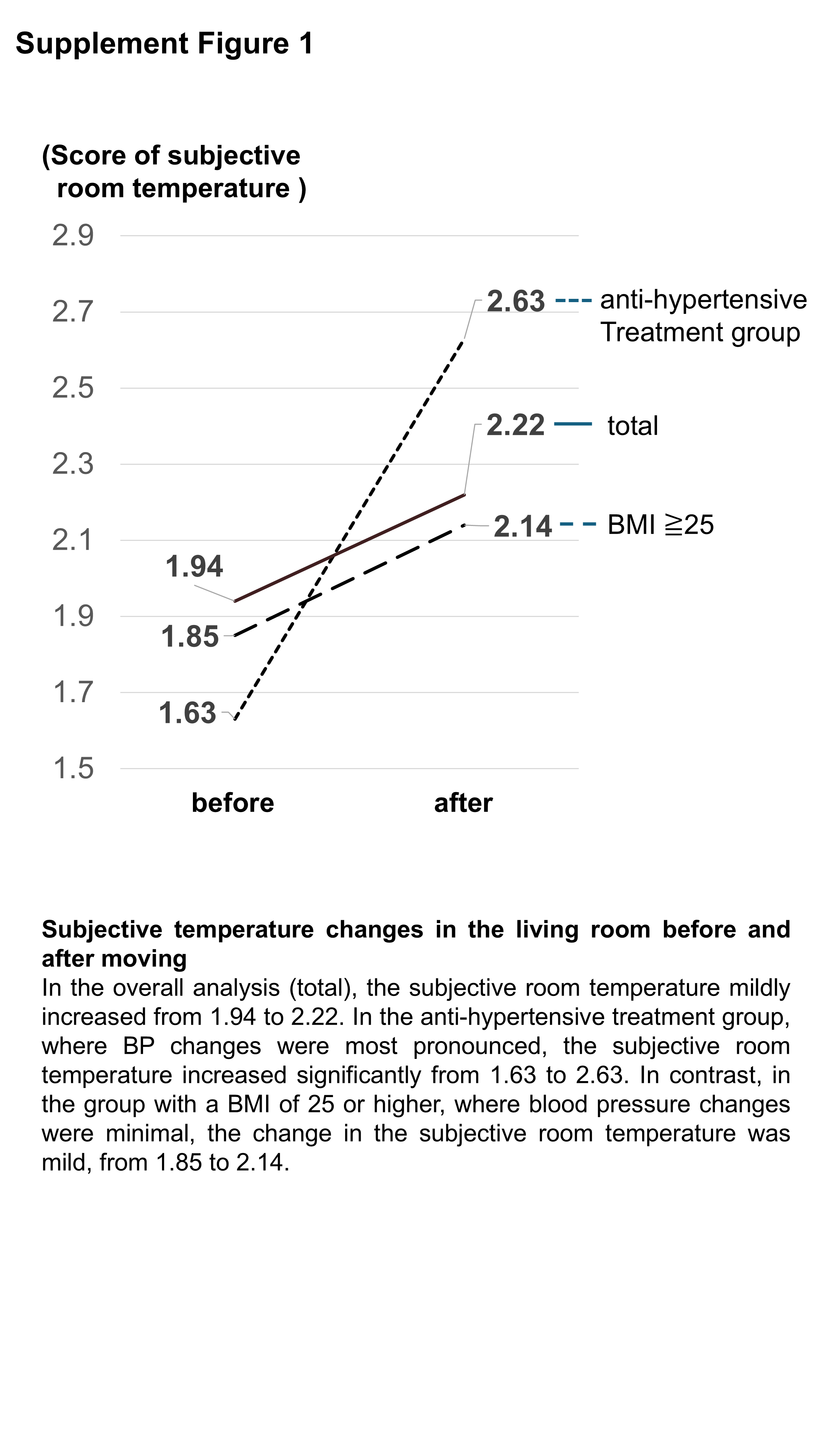

Supplement: Supplementary file 2 — Supplementary Figure 1 [file 41440_2026_2673_MOESM2_ESM.tif]
